# Focal Pancreatic Lesions: Role of Contrast-Enhanced Ultrasonography

**DOI:** 10.3390/diagnostics11060957

**Published:** 2021-05-26

**Authors:** Tommaso Vincenzo Bartolotta, Angelo Randazzo, Eleonora Bruno, Pierpaolo Alongi, Adele Taibbi

**Affiliations:** 1 BiND Department: Biomedicine, Neuroscience and Advanced Diagnostic, University of Palermo, Via Del Vespro, 129, 90127 Palermo, Italy; tommasovincenzo.bartolotta@unipa.it (T.V.B.); angelo.randazzo.90@gmail.com (A.R.); elebru.91@gmail.com (E.B.); taibbiadele@hotmail.com (A.T.); 2 Department of Radiology, Fondazione Istituto Giuseppe Giglio Ct.da Pietrapollastra, Via Pisciotto, Cefalù, 90015 Palermo, Italy; 3 Unit of Nuclear Medicine, Fondazione Istituto Giuseppe Giglio Ct.da Pietrapollastra, Via Pisciotto, Cefalù, 90015 Palermo, Italy

**Keywords:** contrast-enhanced ultrasound, pancreas, diagnostic imaging

## Abstract

The introduction of contrast-enhanced ultrasonography (CEUS) has led to a significant improvement in the diagnostic accuracy of ultrasound in the characterization of a pancreatic mass. CEUS, by using a blood pool contrast agent, can provide dynamic information concerning macro- and micro-circulation of focal lesions and of normal parenchyma, without the use of ionizing radiation. On the basis of personal experience and literature data, the purpose of this article is to describe and discuss CEUS imaging findings of the main solid and cystic pancreatic lesions with varying prevalence.

## 1. Introduction

Contrast-enhanced Ultrasound (CEUS) allows non-invasive assessment of normal and pathologic perfusion of various organs in real time throughout the vascular phase, without the use of ionizing radiation and with a much higher temporal resolution than Computed Tomography (CT) and Magnetic Resonance Imaging (MRI) [1,2,3]. CEUS examination is performed by intravenously injecting Ultrasounds contrast agents (USCAs) consisting of flexible shells (e.g., phospholipids, liposomes) presenting a radius ranging from 1 to 10 μm, containing low solubility gases (e.g., perfluoro-propane, perfluorocarbon, or sulfur hexafluoride) [4]. USCAs microbubbles pass through the lung capillary bed and remain confined within the intravascular space. Approximately 20 min after the injection, the USCAs are completely eliminated: the gas diffuses into the blood and is then exhaled via the pulmonary route, while the shell components are metabolized by the liver or filtered by the kidney [4].

CEUS is safe and well tolerated by patients with hepatic or renal failure, renal obstruction, or chronic obstructive pulmonary disease. In a retrospective analysis of more than 23,000 applications of a sulfur-hexafluoride based contrast agent, 29 cases of mild adverse events were reported and only two cases of anaphylactoid reactions resolved completely without permanent damage [5]. Hence, there is no need of laboratory tests for assessing renal function before administering USCAs.

Contrast-enhanced ultrasonography (CEUS) is not recommended for the detection of a focal pancreatic mass, but it is deemed helpful in the differentiation of lesions such as adenocarcinoma, islet cell tumor, serous cystadenoma or pseudocyst, thus allowing a better patient clinical management [6].

After the detection of a pancreatic mass at US, CEUS should be performed for best accuracy of first line examination before CT and MRI [6].

Furthermore CEUS could reduce the number of false-positive results on MRI for visualization of septa and nodules in pancreatic cystic lesions, so CEUS can be considered a complementary examination for the characterization of cystic pancreatic masses and their follow-up after the initial comprehensive imaging assessment, decreasing the frequency of CT and MRI examinations, limiting radiation and expense [7].

The European Federation of Societies for Ultrasound in Medicine and Biology (EFSUMB) guidelines suggest studying focal pancreatic lesions with CEUS in order to evaluate a pancreatic mass (Table 1), [8].

On the basis of personal experience and literature data, CEUS characteristics of the main solid and cystic pancreatic lesions will be presented and discussed.

## 2. CEUS Technique

CEUS, by using a blood pool contrast agent, can provide dynamic information concerning macro- and micro-circulation of focal lesions and of normal parenchyma. A baseline survey examination with multifrequency convex array probes, including a color/power and pulsed Doppler analysis, was always performed in order to choose the best acoustic window and plane with which to image the lesion, followed by harmonic microbubble specific imaging. The Ultrasound (US) contrast agent, that in our examples used a sulfur hexafluoride-filled microbubble-based contrast agent (SonoVue, Bracco, Milan, Italy), was injected intravenously, followed by 5–10 mL of normal saline flush using a 20- or 22-gauge peripheral intravenous cannula. In order to minimize microbubble disruption, a low frame-rate and a low mechanical index (MI) were used for real-time imaging. Digital cine-loops were registered both during baseline and post-contrast US scanning in the contrastographic phases (early arterial, arterial, pancreatic, portal and late phases). After completion of the pancreatic study, the liver should be assessed in the late phase, exploiting the same contrast injection, in searching for metastases [8].

## 3. Pancreatic Solid Lesions

### 3.1. Pancreatic Ductal Adenocarcinoma

**Figure 1:**
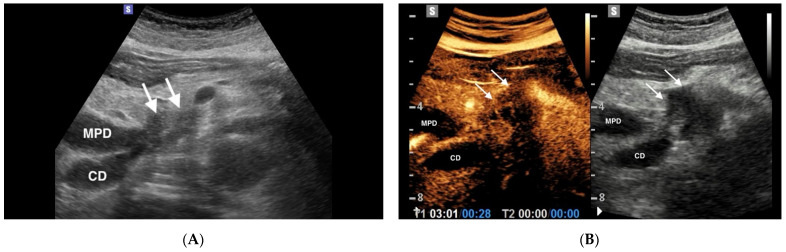
Pancreatic adenocarcinoma in an 89 years old woman. (**A**) B mode Ultrasonography depicts a 3.5 cm heterogenous hypoechoic mass (arrows) in the pancreatic head: severe dilation of the main pancreatic duct (MPD) and cystic duct (CD) is also appreciable. (**B**) On CEUS, in the arterial phase (28 s after the injection of c.a.) the mass is hypoechoic in comparison with the adjacent pancreatic parenchyma (arrows).

Pancreatic cancer is the seventh leading cause of cancer death worldwide, with a total number of deaths in 2020 amounting to 466,003 [9]. Pancreatic ductal adenocarcinoma (PDAC) is the most common primary malignancy of the pancreas, accounting for 85–95% of all pancreatic malignancies [10]. PDAC prognosis is poor, with a 1-year survival rate of less than 20% and a 5-year survival rate of less than 5%. About 70% of PDACs arise in the pancreatic head, while 30% are located in the body/tail. PDAC originates from pancreatic ductal epithelia, with dense cellularity, sparse vascularity, intense stromal desmoplasia and variable necrosis. There are three recognized precursors of invasive PDAC: pancreatic intraepithelial neoplasia (PanIN), intraductal papillary mucinous neoplasm (IPMN) and mucinous cystic neoplasm (MCN) [11]. Adenocarcinoma appears as an ill-defined, heterogeneous hypoechoic mass at US (Figure 1A). Pathologic features of PDAC affect imaging findings. In particular, at CEUS PDAC is typically hypo-enhancing in all phases, because of the desmoplastic reaction with low vascular density (Figure 1B) [12]. On the basis of the hypo-vascular pattern, a multicenter study has reported an accuracy of 87.8% in the characterization of PDAC at CEUS [13]. A recent metanalysis showed a pooled estimate of CEUS sensitivity and specificity in the diagnosis of PDAC of 0.89 (95% CI 0.85–0.92) and 0.84 (95% CI 0.77–0.89), respectively [14]. Of note, in a study encompassing 133 patients, CEUS sensitivity in diagnosing pancreatic ductal adenocarcinoma (86.47%) was reported to be not statistically different from that of multidetector CT (83.58%), *p* = 0.523 [15]. On this basis international guidelines state that in solid pancreatic lesions detected on ultrasound, CEUS can be used to reliably characterize ductal adenocarcinoma [8].

CEUS has been demonstrated to be beneficial for liver staging and to assess relationship with adjacent vessels [7,16]. This information is of paramount importance for preoperative assessment of the resectability of PDAC [17]. When target lesions are not well localized on B-mode ultrasound, CEUS can effectively guide and control percutaneous pancreas biopsies with a percentage of satisfactory percutaneous biopsy as high as 96%, lowering the incidence of complications [18]. The inherent exquisite sensitivity of CEUS in depicting micro-vascularity can be exploited for assessing vascular changes during or after chemotherapy [19].

### 3.2. Pancreatic Neuroendocrine Tumors

Pancreatic neuroendocrine tumors (pNET) represent 1–2% of all pancreatic neoplasms, and about 7% of all NETs [20]. pNETs were previously deemed to originate from the islets of Langerhans (namely, islet cell tumors) but nowadays these tumors are considered to originate from pluripotential stem cells in ductal epithelium [21].

Most pNETs are sporadic, while 10–30% of these occur within hereditary syndrome, such as Multiple Endocrine Neoplasia type I, type IV, neurofibromatosis type I, von Hippel-Lindau disease and tuberous sclerosis. pNETs can be classified into functional and non-functional tumors. About 10% of pNETs are functional with symptoms related to the type of hormone secretion; in this group, insulinomas are the most common (30–40%), followed by gastrinomas (16–30%), glucagonomas (<10%), VIPomas (<10%) and somatostatin-omas (<5%) [22].

About 60–70% of patients with pNETs have a metastatic disease at diagnosis with differences based on histology. Liver metastases are present in more than 50% of patients, strongly influencing the prognosis [23].

**Figure 2:**
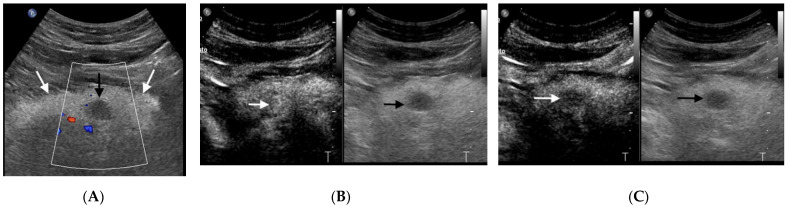
Pancreatic NET: insulinoma. (**A**) Color Doppler Ultrasonography (white box) depicts lack of vascularity within a 1.5 cm hypoechoic mass (black arrow) in the pancreatic body (white arrows). (**B**) On CEUS, in the arterial phase (19 s after the injection of c.a.), the mass is slightly hyperechoic in comparison with the adjacent pancreatic parenchyma (arrow), indicating rich vascularity. (**C**) On CEUS, in the late phase (120 s after the injection of c.a.), the mass is hypoechoic in comparison with the adjacent pancreatic parenchyma (arrow), indicating “wash-out”.

On CEUS, pNETs usually appear as hyper-enhancing lesions in the arterial phase, either homogeneous or heterogeneous, owing to their abundant arterialization (Figure 2) [13]. The presence of heterogeneous enhancement is often related to the presence of necrotic areas within the lesion, especially in larger tumors.

According to the European Federation of Societies of Ultrasound in Medicine and Biology (EFSUMB) and the European Neuroendocrine Tumor Society (ENETS) consensus guidelines, CEUS can be used to distinguish between pancreatic ductal adenocarcinoma and neuroendocrine tumors (Table 2) [8,23].

## 4. Neoplastic Cystic Lesions

Pancreatic cystic lesions (PCLs) encompass a broad spectrum of entities with different malignant potential. PCLs can be categorized into neoplastic and nonneoplastic. Common cystic neoplasms include intraductal papillary mucinous neoplasm (IPMN), mucinous cysto-adenoma and serous cysto-adenoma. Uncommon cystic neoplasms are represented by solid pseudopapillary tumor, neuroendocrine cystic tumor and cystic adenocarcinoma.

IPMNs, mucinous cystic neoplasms (MCNs), and solid pseudopapillary neoplasms (SPNs) can undergo malignant transformation, whereas cystic pancreatic neuroendocrine tumors (cNETs) already harbor metastatic potential. On the other hand, nonneoplastic PCLs, such as pseudocysts, lymphoepithelial cysts, and retention cysts, behave indolently and almost never progress into malignancy [24]. Therefore, the evaluation of a PCL should lead to the differentiation between benign and malignant nature of the lesion, thus prompting the correct management strategy [25].

PCLs are an increasingly recognized clinical entity: in asymptomatic patients, up to 2.5% are found to have pancreatic cysts, a number that increases to 10% of patients older than 70 years. The frequency of incidentally detected PCLs in asymptomatic patients is increasing and is currently estimated to represent more than 60% of all detected cystic lesions in the pancreas [26]. Prevalence, size and number of lesions per patient increase with age but the widespread use of cross-sectional imaging has led to a surge of PCL prevalence. An almost 8% linear increase in prevalence between 1995 and 2010 has been reported, along with a simultaneous decrease in cyst size from a mean of 2.4 cm between the years 1995 and 2005 to a mean of 1.6 cm between 2005 and 2010 [27]. Incidental PCLs are discovered in 1.2–2.6% of CT scans, 2.4–19.9% of MRI scans and up to 45% in MRCP studies [28,29,30,31].

The surge of incidentally detected PCLs by cross-sectional imaging has led to the definition “disease of technology” and to the clinical conundrum of a proper management. Several international associations have prompted white papers, recommendations and guidelines to manage PCLs, in terms of diagnosis, imaging surveillance, performance, and cost-effectiveness, but there is no definitive strategy for the differentiation between the various types of PCN and for neoplastic grading. Yet, given the low rate of malignant transformation of pancreatic cysts (0.12% annually), a “low intensity” surveillance strategy may be considered appropriate for PCLs without high-risk or worrisome features at imaging (Table 3) [32,33,34,35,36].

### 4.1. Mucinous Cystic Neoplasms

Cystic mucin-producing pancreatic neoplasms are classified by the World Health Organization (WHO) into two distinct entities, based on the presence of ovarian stroma: intraductal papillary mucinous neoplasms (IPMNs) and mucinous cystic neoplasms (MCNs) [37].

#### 4.1.1. IPMN

Described for the first time in 1982 by Ohhashi K et al. as neoplasm with mucin hyperproduction, dilation of the duct of Wirsung and protruding papilla (the Ohhashi triad), intraductal papillary mucinous neoplasms (IPMNs) were more specifically described in 1996 by the WHO as: “neoplasm covered with columnar cells containing high mucin with or without papillary projections, involving the main pancreatic duct and/or secondary ducts without an ovarian stroma”.

There are four subtypes of IPMN: Gastric, Intestinal, Oncocitic, and Pancreatobiliary. All subtypes progress via the classic adenoma–carcinoma sequence [26]. IPMNs communicate with the ductal system and can be further classified into main duct (MD), branch duct (BD), or mixed type IPMNs [38]. This latter classification is of clinical relevance, considering that in terms of malignant potential, MD-IPMNs and mixed IPMNs have greater risk (36–100%) compared to BD-IPMNs (11–30%) [39].

The majority (70%) of IPMNs present in the head of the pancreas, but 20% present in the body or tail. Unlike other common PCNs, which all present as solitary cysts, IPMNs can be solitary or multifocal. In the MD-IPMN, US may demonstrate involvement of the main pancreatic duct when it is dilated, and the IPMN as a hypo-anecoic mass downstream of the main pancreatic duct dilatation [40]. Dilatation > 1 cm of main duct is suggestive for IPMN of main duct; a pancreatic mucinous cyst communicating with the pancreatic duct without main duct dilation is suggestive for IPMN of branch duct [41]. Unfortunately, neither US nor CEUS are able to effectively demonstrate communication with the ductal system, which is essential for the final diagnosis of branch duct IPMN. On the other hand, CEUS can easily demonstrate the presence of vascularized mural nodules and tumoral vegetations within a pancreatic cyst and differentiate viable tumoral tissue from avascular mucin plug areas [1,42]. For this purpose, D’Onofrio et al. showed that there are no significant differences in diagnostic accuracy between CEUS and MRI in the identification of septa and nodules [7].

**Figure 3:**
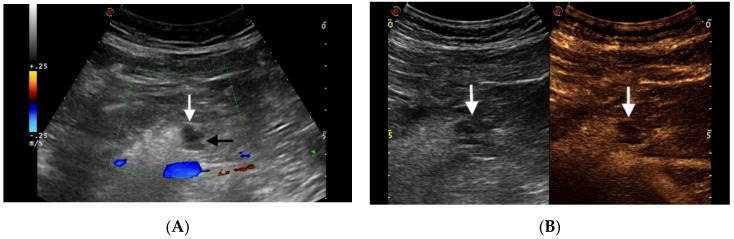
Branch duct IPMN. (**A**) Color Doppler Ultrasonography (dotted box) depicts lack of vascularity within a 1 cm anechoic cystic mass (white arrow) in the pancreatic body. A tiny septum is also appreciable within the lesion (black arrow). (**B**) On CEUS the lesion shows lack of vascularity throughout the different phases, as well as the internal septum (arrows).

The differential diagnosis of these cystic lesions ranges from benign to potentially or truly malignant lesions. The most common are considered benign, particularly those that are small in size, but they have the potential to become malignant. Size of cyst > 3cm, solid component associated with the cyst, and dilated pancreatic duct are the main imaging features of pancreatic cysts predictive of risk of malignancy according to the American Gastroenterological Association guidelines [43]. Some cystic lesions present similar morphologic features, which make preoperative imaging diagnosis difficult. CEUS’s capability to detect septa and nodules can contribute to the differentiation of a cystic neoplasm from nontumoral cyst, as well as the determination of their possible malignant potential (Figure 3).

#### 4.1.2. MCNs

Mucinous cystic neoplasms (MCNs) represent about 10% of all PCLs and include mucinous neoplasms with low-grade dysplasia (MCN) and mucinous cystadenocarcinoma. MCNs occur almost exclusively in middle-aged females and the median age of diagnosis is mid-to-late 40s [44]. They are usually solitary and are located in the tail and body of the pancreas (90–95%) [45]. MCNs are usually large, septated, thick-walled mucinous uni- or oligo-locular cysts that lack communication with the ductal system and occur in middle-aged women. The most characteristic histological finding in MCNs is the presence of a unique ovarian-type stroma not found in other pancreatic neoplasms [45]. MCNs may show peripheral calcifications [24]. Increased cystic fluid Carcino-Embryogenic Antigen (CEA) level can be helpful in discerning MCNs from other non-mucinous cystic lesions.

At imaging, including ultrasonography, MCNs usually show round morphology, sharp margin, thick wall, septa, and fluid content, often particulate. The presence of mural nodules and peripheral calcifications are imaging findings in favor of mucinous cysto-adeno-carcinoma. US may depict a uni- or oligolocular cystic mass with mural or septal nodules and irregular thickening of the cystic wall [40]. CEUS may depict enhancement of septa, nodules and cystic wall, during early pancreatic phase and hypo-enhancement during the delayed phase, thus indicating a malignant lesion as cystadenocarcinoma [46]. For this purpose, CEUS compares favorably with MRI in displaying the inner structure of these cystic neoplasms and can provide useful clues for diagnosis [47].

MCN is considered a pre-malignant lesion and surgical resection for all surgically fit candidates with MCNs is recommended by several international consensus guidelines [32].

### 4.2. Serous Cystic Neoplasms

Serous cystic neoplasms (SCNs) represent less than 1% of all primary pancreatic lesions and about 30% of all cystic neoplasms of the pancreas [48]. SCNs are benign cystic tumors of the pancreas and represent approximately 16% of resected PCLs. These tumors occur more frequently in women (75%) at a mean age of 50 to 60 years [44]. SCNs grow slowly and rare case reports document serous cystadenocarcinomas. As a consequence, surgery is usually reserved for symptomatic patients.

Serous cystadenoma (SCA) is usually solitary, without communication with the main pancreatic duct. Typically, it presents a multilocular honeycomb architecture due to the presence of multiple micro-cysts (<20 mm), thin wall and thin multiple septa orientated toward the center [24]. Of note, oligo-macro-cystic appearance of SCA may be encountered. Macro-cystic type (25%), especially the unilocular type, may be difficult to be differentiated from MCA [49]. In contrast to MCNs, SCA may contain central, stellate calcification and in 15% of cases a central scar may be present [50].

SCAs are typically hyper-enhancing on CEUS, since the septa are composed of abundant sub-epithelial micro- and macro-vessels and, especially when the cysts are small, they may mimic a solid lesion [1]. CEUS improves the US characterization of SCA, showing the enhancement of the centrally oriented septa with better identification of the “honeycomb” multilocular architecture of the lesion [51].

CEUS may be helpful not only in the differential diagnosis of SCA, but also in the long-term follow-up of these tumors, which can be conservatively managed in most cases.

## 5. Non Neoplastic Cystic Lesions

### Pseudocyst

Pseudocysts are defined as a collection of amylase-rich fluid that contains debris, blood, and inflammatory cells, and is surrounded by a fibrous wall with no epithelial lining [24]. Pseudocysts are the most common pancreatic nonneoplastic cystic lesions and they occur after episodes of acute pancreatitis, or are superimposed on chronic pancreatitis due to alcoholic, biliary, or traumatic cause [52]. Pseudocysts do not enhance at any phase with CEUS, even when heterogeneous on B-mode US [1,42]. The reported sensitivity and specificity of CEUS in characterizing pseudocysts is up to 100% [46]. EFSUMB guidelines state that CEUS can be used to differentiate between cystic neoplasms and pseudocysts [8].

## 6. Chronic Pancreatitis

**Figure 4:**
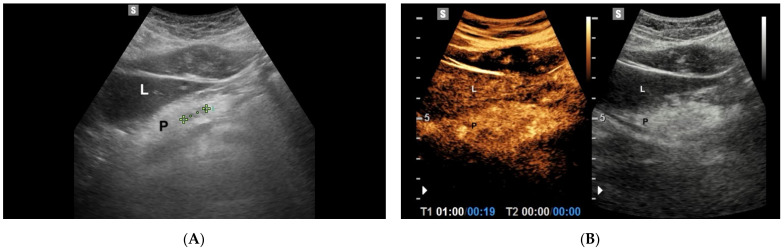
Focal autoimmune pancreatitis. (**A**) B mode Ultrasonography shows a 1.2 cm hypoechoic lesion (calipers) in the pancreatic body; (L: liver; P: Pancreas). (**B**) On CEUS, in the arterial phase (19 s after the injection of c.a.), the lesion is isoechoic in comparison with the adjacent pancreatic parenchyma.

Chronic pancreatitis (CP) is characterized by fibrosis, destruction and distortion of the pancreatic ducts with loss of parenchyma. The most important diagnostic sign of chronic pancreatitis is the presence of calcifications and the dilatation (more than 3 mm) or irregular course of the pancreatic duct [53,54]. During the evolution of CP, inflammatory masses can appear, a characteristic feature of pseudo-tumoral CP. Differential diagnosis between this entity and pancreatic cancer is often difficult due to the similar imaging aspect: they, indeed, are both hypoechoic in conventional US [55]. CEUS can improve the differential diagnosis between pseudo-tumoral CP and pancreatic adenocarcinoma. D’Onofrio et al. showed the hypoecho-genicity of ductal adenocarcinoma in all contrast-enhanced phases, due to its intense desmoplastic reaction with poor mean vascular density of the lesion, contrary to the enhancement in the early contrast-enhanced phase characteristic of the inflammatory mass (Figure 4) [56].

The inflammatory origin of the lesion is therefore supported by the presence of parenchymal enhancement similar to that of the adjacent pancreas during the dynamic study, and the intensity of the enhancement is related to the length of the inflammatory process. However, in long-standing chronic inflammatory processes, inhomogeneous hypo-vascularization of the lesion may be observed, probably owing to the presence of a large amount of fibrosis, and differential diagnosis becomes more difficult [33,57].

## 7. Conclusions

CEUS has been proved to be an accurate imaging method for evaluating differences in the vascularity of various pancreatic lesions and helpful in the differentiation between solid and cystic lesions. CEUS is a robust and safe technique which can allow an immediate characterization of the lesion or its follow-up.

## Figures and Tables

**Table 1:** EFSUMB Recommendation for studying focal pancreatic lesion on CEUS.

EFSUMB Recommendation for CEUS
Characterization of ductal adenocarcinoma for lesions showed on US Differential diagnosis between ductal adenocarcinoma and neuroendocrine tumors Differential diagnosis between pseudocysts and cystic tumors Differentiation of vascular (solid) from avascular (liquid/necrotic) components of a lesion Defining the dimensions and margins of a lesion, including its relationship with adjacent vessels Characterization of acute necrotizing pancreatitis Improving accuracy of percutaneous ultrasound-guided pancreatic procedures Diagnosis of cystic lesions that are indeterminate on CT

**Table 2:** Features and enhancing pattern of focal pancreatic lesions in CEUS.

	Hypo-Vascular Heterogeneous	Hyper-Vascular Heterogeneous	Iso-Vascular Heterogeneous	Hyper-Vascular Homogeneous	Hypo-Vascular Homogeneous	Iso-Vascular Homogeneous
**Solid**	Adenocarcinoma	Neuroendocrine tumors	Adenocarcinoma	Neuroendocrine tumors	Adenocarcinoma	Adenocarcinoma
		Adenocarcinoma		Adenocarcinoma		
**Cystic simple**	-	Neuroendocrine tumors	Mucinous cystadenoma	Neuroendocrine tumors	Pseudocyst	Pseudocyst
					IPMN ^1^	IPMN
					Serous cystadenoma	Serous cystadenoma
**Cystic with septa**	Adenocarcinoma	-	Adenocarcinoma	-	Adenocarcinoma	Adenocarcinoma
			Mucinous cystadenoma		IPMN	IPMN
					Serous cystadenoma	Serous cystadenoma
**Cystic with nodules**	Cystadenocarcinoma	Cystadenocarcinoma	Cystadenocarcinoma	Neuroendocrine tumors	Cystadenocarcinoma	Mucinous cystadenoma
	Mucinous cystadenoma	Neuroendocrine tumors	Mucinous cystadenoma	Cystadenocarcinoma	Mucinous cystadenoma	
**Cystic with thick wall**	IPMN	Neuroendocrine tumors	IPMN	Neuroendocrine tumors	IPMN	IPMN
			Pseudocyst		Pseudocyst	Pseudocyst
	Mucinous cystadenoma		Serous cystadenoma	Adenocarcinoma	Serous cystadenoma	Serous cystadenoma
	Adenocarcinoma		Mucinous cystadenoma		Mucinous cystadenoma	Mucinous cystadenoma
**Cystic with septa, nodules and thick wall**	IPMN	Neuroendocrine tumors	IPMN	Neuroendocrine tumors	IPMN	IPMN
	Adenocarcinoma		Adenocarcinoma		Adenocarcinoma	Adenocarcinoma
	Cystadenocarcinoma		Cystadenocarcinoma		Cystadenocarcinoma	

^1^ IPMN: Intraductal papillary mucinous neoplasms.

**Table 3:** Pancreatic Cystic Lesions: Imaging features in favor of malignancy.

Pancreatic Cystic Lesions: Imaging Features in Favor of Malignancy
“High-risk stigmata” ○ main pancreatic duct diameter of at least 10 mm ○ obstructive jaundice associated with a cyst in the pancreatic head ○ solid enhancing nodular lesion within the cyst “Worrisome features” ○ cyst ≥ 3 cm ○ thick or contrast-enhancing cyst walls ○ main pancreatic duct diameter of 5–9 mm ○ non-enhancing mural nodule
